# A Simple Effective Pharmacological Treatment of Hypoxemia During One-Lung Ventilation

**DOI:** 10.3390/jcm8081139

**Published:** 2019-07-31

**Authors:** Davide Chiumello, Silvia Coppola, Erica Ferrari

**Affiliations:** 1 Department of Anesthesia and Intensive Care, ASST Santi Paolo e Carlo, San Paolo University Hospital, 20142 Milan, Italy; 2 Department of Health Sciences, University of Milan, 20142 Milan, Italy; 3 Coordinated Research Center on Respiratory Failure, University of Milan, 20123 Milan, Italy; 4 Department of Pathophysiology and Transplantation, University of Milan, 20123 Milan, Italy

**Keywords:** iloprost, hypoxemia, one-lung ventilation

## Abstract

Hypoxemia during one-lung ventilation is a challenge in the clinical practice. Moving from the results of the study conducted by Choi et al., we discuss the possibility to modulate hypoxemia by administering iloprost via inhalation, in the light of the physiological mechanisms.

Currently, the common indications for one-lung ventilation include thoracic, esophageal surgery and the need to deliver a one-lung ventilation in case of massive hemorrhage, lung lavage, or bronchopleural fistula [1]. In lateral position, during double ventilation in sedated and paralyzed patients the dependent lung is partially collapsed by the displacement of the diaphragm and by the weight of the mediastinum. Due to the higher perfusion in these regions, the ventilation perfusion mismatch can be increased. In fact, the blood flow that can perfuse the dependent lung is the 60% of the cardiac output compared to 40% in the non-dependent lung [2]. In double lung ventilation in lateral position, assuming an equal amount of shunt, 5% for each lung, the percentage of cardiac output that will participate in gas exchange will be 55% and 35% for the dependent and non-dependent lung, respectively.

During one-lung ventilation in lateral position, due to the absence of any ventilation with the persistence of perfusion in the non-dependent lung (i.e., theoretically, an increase in total shunt of 35%), hypoxemia can develop. The larger the size of the non-ventilated lung, the larger the size of the shunt. Additional factors such as the changes in cardiac output, the increase in pulmonary vascular resistance, the decrease in hemoglobin level, the double lumen malposition, and the type of anesthetic agent can affect the arterial oxygenation [3,4,5,6]. The duration of surgery, the possible generation of lung edema, and the absorption atelectasis in the dependent lung will contribute to hypoxemia. 

Moderate hypoxemia, defined as an arterial oxygen saturation ranging between 85% and 90%, has been reported in 5–10% of the patients undergoing one-lung ventilation [1,7]. To mitigate the effect of the hypoxemia, the onset of hypoxemic pulmonary vasoconstriction via a reduction in the blood perfusion in the non-dependent lung and an increase in the perfusion in the dependent lung [4] should ameliorate the ventilation–perfusion ratio. The possible prevention and treatment of hypoxemia during one-lung ventilation includes the delivery of an adequate ventilation in the dependent lung with a certain level of Positive End Expiratory Pressure, the oxygen administration in the non-dependent lung, a sufficient level of hemoglobin, and the possibility to modulate the lung perfusion (i.e., change the ventilation–perfusion ratio) [1,3,4]. Previous studies have evaluated the possibility to increase the perfusion in the ventilated regions by using nitric oxide or reducing the perfusion in the non-ventilated regions by almitrine [4,8,9]. Based on these data, the inhaled iloprost, which is a prostacyclin analogue, by a selective vasodilation of the pulmonary vascular bed, could improve the arterial oxygenation during one-lung ventilation, thereby ameliorating the ventilation–perfusion ratio.

In this issue of the Journal, Choi et al. investigated the effects of inhaled iloprost on oxygenation during one-lung ventilation in patients undergoing lung surgery. Seventy-two patients with non-small cell lung cancer scheduled for elective video-assisted thoracoscopic lobectomy were enrolled [10]. Patients were randomized to one of three groups: control group (distilled water), iloprost10 (iloprost 10 μg), and iloprost20 (iloprost 20 μg). Mechanical ventilation was delivered with a tidal volume of 6 mL/kg and respiratory rate was adjusted to maintain a partial pressure of carbon dioxide in the arterial blood within 35–45 mmHg, with an oxygen fraction of 100%. The solution was administered by a jet nebulizer connected to the Y piece of the ventilator inspiratory line with an oxygen flow of 6 L/min. The total amount of dose reaching the lung was 2.5 and 5.0 μg in the two iloprost groups, respectively. Arterial oxygenation and shunt were respectively significantly higher and lower in the iloprost20 compared both to the iloprost10 group and to the control group. In the iloprost10 group and the control group, the arterial oxygenation was significantly lower during one-lung ventilation compared to double-lung ventilation. The other hemodynamic parameters were similar among the three groups. Based on the data, the authors suggest the use of iloprost inhalation via the jet nebulized to prevent hypoxemia during one-lung ventilation in thoracic surgery. Iloprost, when given by inhalation in a correct dose, should vasodilate the pulmonary circulation without any major side effects on the systemic circulation. 

The main beneficial effects of the inhaled iloprost, which mostly locally vasodilates the pulmonary vessels, are the reduction of pulmonary arterial pressure and the increase in the perfusion of these lung regions [4]. Based on these properties, the inhaled iloprost has been found to reduce the pulmonary arterial pressure and to improve the oxygenation in patients with acute respiratory failure [11,12,13]. Similar to Acute Respiratory Distress Syndrome patients characterized by the simultaneous presence of collapsed and normally ventilated regions, healthy patients in lateral position during one-lung ventilation present a totally unventilated non-dependent lung and collapsed regions still perfused in the dependent lung. Inhaled iloprost seems to have beneficial effects on the oxygenation, which is impaired during one-lung ventilation in lateral position, without hypotension or other major deleterious hemodynamic effects. Contrary to the previous data on nitric oxide administration (another powerful pulmonary vasodilator, which had controversial effects on the arterial oxygenation), iloprost significantly improved the oxygenation [8,9]. Regarding the optimum dose of inhaled iloprost to use for pulmonary vasodilation, the present data suggest an administration of 20 μg once, with a persistent effect for the entire duration of the surgery.

Although these data arise from a single center randomized study enrolling a small number of patients, the anesthesiologist should be reminded of the possibility to use inhaled iloprost in case of hypoxemia during thoracic surgery that does not respond to classical ventilatory management, such as PEEP, recruitment, continuous positive airways pressure, and high oxygen fraction.
